# Postprostatectomy Radiotherapy Timing and Long-Term Health-Related Quality of Life

**DOI:** 10.1001/jamanetworkopen.2024.40747

**Published:** 2024-10-24

**Authors:** Sagar A. Patel, Dattatraya Patil, Joseph Smith, Christopher S. Saigal, Mark S. Litwin, Jim C. Hu, Matthew R. Cooperberg, Peter R. Carroll, Eric A. Klein, Adam S. Kibel, Gerald L. Andriole, Misop Han, Jeff M. Michalski, David P. Wood, Larry A. Hembroff, Daniel E. Spratt, John T. Wei, Howard M. Sandler, Daniel A. Hamstra, Louis Pisters, Deborah Kuban, Meredith M. Regan, Andrew Wagner, Catrina M. Crociani, Irving Kaplan, Martin G. Sanda, Peter Chang

**Affiliations:** 1 Department of Radiation Oncology, Emory University, Atlanta, Georgia; 2 Department of Urology, Emory University, Atlanta, Georgia; 3 Department of Urology, Vanderbilt University, Nashville, Tennessee; 4 Department of Urology, University of California, Los Angeles; 5 Department of Urology, Weill Cornell Medical College, New York, New York; 6 Department of Urology, University of California, San Francisco; 7 Glickman Urological and Kidney Institute, Cleveland Clinic, Cleveland, Ohio; 8 Department of Urology, Brigham and Women’s Hospital, Boston, Massachusetts; 9 Department of Urology, Johns Hopkins University, Baltimore, Maryland; 10 Department of Radiation Oncology, Washington University in St Louis, St Louis, Missouri; 11 Department of Urology, Beaumont Health, Royal Oak, Michigan; 12 Office of Survey Research, Michigan State University, East Lansing; 13 Department of Radiation Oncology, Case Western Reserve University, Cleveland, Ohio; 14 Department of Urology, University of Michigan, Ann Arbor; 15 Department of Radiation Oncology, Cedars Sinai Medical Center, Los Angeles, California; 16 Department of Radiation Oncology, Baylor College of Medicine, Houston, Texas; 17 Department of Urology, The University of Texas MD Anderson Cancer Center, Houston; 18 Department of Radiation Oncology, The University of Texas MD Anderson Cancer Center, Houston; 19 Department of Biostatistics and Computational Biology, Dana Farber Cancer Institute, Boston, Massachusetts; 20 Department of Surgery, Beth Israel Deaconess Medical Center, Boston, Massachusetts; 21 Department of Radiation Oncology, Beth Israel Deaconess Medical Center, Boston, Massachusetts

## Abstract

**Importance:**

The association between radiotherapy (RT) timing after radical prostatectomy and long-term patient-reported health-related quality of life (HRQOL) in men with prostate cancer is unknown.

**Objective:**

To measure long-term HRQOL in men with prostate cancer up to 15 years after prostatectomy with or without RT and examine whether early vs late postprostatectomy RT is associated with differences in sexual, urinary, and bowel HRQOL.

**Design, Setting, and Participants:**

A prospective, multicenter, longitudinal cohort analysis using HRQOL data from the PROST-QA (2003-2006) and RP2 consortium (2010-2013) studies was conducted. Men with localized prostate cancer undergoing radical prostatectomy were included. Data were analyzed between May 8, 2023, and March 1, 2024. The study was conducted in 12 high-volume academic medical centers in the US.

**Exposures:**

Men were stratified based on receipt and timing of postprostatectomy RT: prostatectomy only, early RT (<12 months), and late RT (≥12 months).

**Main Outcomes and Measures:**

Longitudinal sexual, incontinence, urinary irritation, bowel, and hormonal/vitality HRQOL were measured via the Expanded Prostate Cancer Index Composite at baseline; months 2, 6, and 12; and annually thereafter. Treatment groups were compared using multivariable linear mixed-effects models of change in longitudinal domain scores. Pad use for incontinence was measured longitudinally among men receiving postprostatectomy RT.

**Results:**

A total of 1203 men were included in the study: prostatectomy only (n = 1082), early RT (n = 57), and late RT (n = 64). Median age for the entire cohort was 60.5 (range, 38.8-79.7) years, and 1075 men (92.0%) were White. Median follow-up was 85.6 (IQR, 35.8-117.2) months. Compared with men receiving prostatectomy alone, those receiving postprostatectomy RT had significantly greater decreases in sexual, incontinence, and urinary irritation HRQOL. However, timing of postprostatectomy RT, specifically early vs late, was not associated with a long-term decrease in any HRQOL domain. There was evidence of improved recovery of sexual, continence, and urinary irritation scores among men receiving early RT compared with those receiving late RT after prostatectomy. Before the start of postprostatectomy RT, 39.3% of men in the early RT cohort and 73.4% of men in the late RT cohort were pad-free. By the sixth visit post-RT, 67.4% in the early RT cohort and 47.6% in the late RT cohort were pad-free.

**Conclusions and Relevance:**

In this multicenter, prospective analysis, postprostatectomy RT appeared to be negatively associated with long-term HRQOL across all domains. However, receipt of early vs late postprostatectomy RT may result in similar long-term HRQOL outcomes.

## Key Points

**Question:**

Is radiotherapy timing after radical prostatectomy associated with long-term patient-reported health-related quality-of-life (HRQOL) in men with prostate cancer?

**Findings:**

In this cohort study of 1203 men with localized prostate cancer undergoing initial prostatectomy, receipt of postprostatectomy radiotherapy was associated with statistically significant long-term decrements in patient-reported urinary incontinence, urinary irritation, bowel, and sexual HRQOL. However, long-term HRQOL did not significantly differ between men receiving early (<12 months) vs late (≥12 months) radiotherapy after prostatectomy.

**Meaning:**

The findings of this study suggest that receipt of early vs late radiotherapy following radical prostatectomy may result in similar long-term patient-reported outcomes.

## Teaser

This cohort study examines health-related quality of life in men with prostate cancer who have undergone prostatectomy only vs prostatectomy with radiotherapy within or beyond 1 year following surgery.

## Introduction

Radical prostatectomy use in men with localized, high-risk prostate cancer is increasing.^1^ While the procedure is curative for some, a substantial proportion of men will experience prostate-specific antigen (PSA) persistence or a delayed increase in PSA, a situation known as biochemical recurrence. For these men, half will develop metastatic progression within 5 to 10 years without salvage therapy.^2^

Three historic randomized trials showed that adjuvant radiotherapy (RT) to the prostate bed vs surveillance alone following radical prostatectomy in men with high-risk pathologic features or positive surgical margins significantly decreases the risk of progression and possibly distant metastases.^3,4,5^ These trials, however, were conducted in an era before routine use of postprostatectomy PSA monitoring. Subsequently, 3 randomized clinical trials^6,7,8^ and a meta-analysis^9^ conducted when postprostatectomy PSA monitoring was routine compared adjuvant (ie, soon after surgery, when the PSA is undetectable) vs early (ie, delayed after surgery, at the time of biochemical recurrence) salvage RT and found no significant difference in progression-free survival between these 2 approaches. Adjuvant RT, however, was associated with worse genitourinary and erectile toxic effects.^6,7,8^ As such, these trials have led to a widespread uptake of PSA-guided salvage, as opposed to adjuvant, RT after prostatectomy.

However, men with adverse pathologic features were underrepresented in these 3 recent trials, as those with a Gleason score of 8 to 10 and/or pT3-T4 category comprised, at most, 9% to 17% of enrolled men.^6,7,8^ Whether delaying RT at the time of biochemical recurrence results in noninferior outcomes in this high-risk subgroup remains unknown. For example, one international multicenter cohort and one US multicenter cohort each showed a significant improvement in all-cause mortality with adjuvant vs early salvage RT for men with high-risk pathologic factors after prostatectomy.^10,11^

A primary impetus for delaying RT after prostatectomy is mitigating genitourinary and erectile toxic effects, yet few studies, if any, have rigorously compared long-term patient-reported health-related quality of life (HRQOL) outcomes based on timing of postprostatectomy RT. Herein, we compare 15-year patient-reported outcomes following early vs late postprostatectomy RT within the PROST-QA (PQA) and PROSTQA-RP2 (RP2) multicenter, prospective cohorts.

## Methods

### Study Participants

Men with previously untreated clinical category T1-T2 prostate cancer pursuing upfront radical prostatectomy were analyzed from 2 prospective, multicenter cohorts: PQA and RP2 (eTable 1 in Supplement 1). The PQA and RP2 consortiums each had 9 academic hospitals; collectively, however, they have 12 individual hospitals (as many participating hospitals overlapped between the 2). The PQA consortium has been previously described,^12^ with patients accrued between 2003 and 2006. From the 1201 participants who underwent definitive prostate cancer treatment in PQA, 602 men underwent initial prostatectomy with or without postprostatectomy RT and were eligible for this analysis. The RP2 cohort also comprised 9 academic hospitals, with patients accrued between 2010 and 2013. Of the 677 participants in the RP2 cohort, 601 underwent initial prostatectomy with or without postprostatectomy RT and were eligible for this analysis. The final analytic cohort consisted of 1203 men. Because the study used deidentified data, the requirement for formal institutional review and the need for informed consent were waived, consistent with the policies of Emory University School of Medicine. The study followed the Strengthening the Reporting of Observational Studies in Epidemiology (STROBE) reporting guideline for cohort studies. All data analyses were conducted from May 8, 2023, through March 1, 2024.

**Figure 1.**
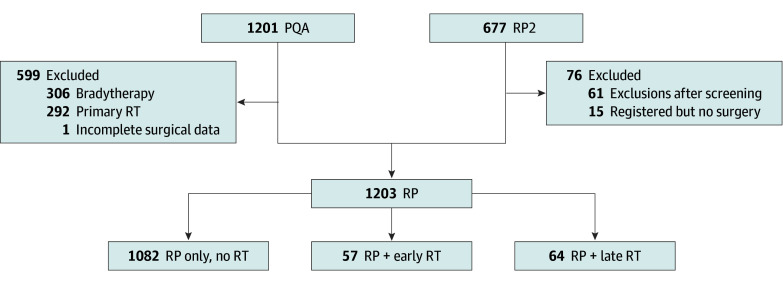
Flowchart of Eligible PROST-QA (PQA) and PROSTQA-RP2 (RP2) Comparative Groups RP indicates radical prostatectomy; RT, radiotherapy (early, <12 months after radical prostatectomy; late, ≥12 months after radical prostatectomy).

Participants were stratified based on receipt and timing (early vs late) of postprostatectomy RT. Early RT was defined as less than 12 months after robotic-assisted laparoscopic prostatectomy, and late RT was defined as 12 months or more after robotic-assisted laparoscopic prostatectomy (Figure 1). All patients undergoing postprostatectomy RT received treatment at the same facility as surgery.

### Outcomes Assessment

Measures of HRQOL were collected using the Expanded Prostate Cancer Index Composite (EPIC-26)^13^ by a third-party telephone survey facility at pretreatment baseline (ie, before prostatectomy); at 2, 6, and 12 months; and annually thereafter after the initial prostatectomy. For men who received postprostatectomy RT, a change in domain scores from immediately before RT to long-term follow-up after completion of RT was also assessed. In addition, the proportion of patients free from using protective urinary pads at each follow-up time point following completion of RT was measured.

### Statistical Analysis

Differences in clinicodemographic characteristics between men who received no RT, early RT, and delayed RT were compared using the χ^2^ or Fisher exact test for categorical covariates and the Wilcoxon rank-sum test for continuous covariates. Scores for patient-reported outcomes, as measured by EPIC-26, were calculated as described previously.^14,15,16^ Domain scores are on a 0- to 100-point scale, representing worse to favorable related QOL. To assess the receipt and timing of postprostatectomy RT on HRQOL over time, linear mixed-effects models were fitted for each HRQOL domain after variance-covariance structure selection through the Akaike information criterion and model diagnosis. These models accounted for correlation in repeated measurements, interaction with time indicator, and random effect of participating study (PQA vs RP2) for which the institutions and time periods of enrollment differed. Postprostatectomy RT timing (ie, early or late) and concomitant androgen deprivation therapy (ADT) use with RT were a priori included in the model, irrespective of their significance, while adjusting for baseline HRQOL domain score, age, self-reported race, educational level, cohabitation, prostate volume, Gleason score, tumor category, baseline PSA, comorbidities, body mass index, and use of nerve-sparing surgery. Prostate cancer risk was defined using initial T category, PSA, and Gleason score as follows: low risk (PSA score <10, Gleason score <7, and T1), high risk (Gleason score >7 or PSA score >20), and intermediate risk (all others who do not meet criteria for low or high risk). Backward selection was used until a parsimonious model was reached. A clinically relevant change in the HRQOL domain (ie, minimally important difference) was defined as a difference from baseline to follow-up that exceeded half an SD of the baseline preprostatectomy value.^17^ To further assess postprostatectomy RT and HRQOL, we analyzed change in HRQOL domain score from the last available postprostatectomy and pre-RT visit to the last follow-up, restricted to patients who received postprostatectomy RT. This analysis shifted the baseline (ie, time 0) to the visit immediately before the start of RT, while using the same analytical method described above. All statistical analyses were performed using SAS, version 9.4 (SAS Institute Inc) with a significance level determined with 2-sided, paired testing of *P* < .05.

## Results

### Study Participants

Among the eligible 1203 men for this analysis, 1082 underwent prostatectomy alone, 57 underwent prostatectomy and early RT, and 64 underwent prostatectomy and late RT. Patient attrition and interview completion rates over the 15-year follow-up period are reported in eTable 2 in Supplement 1. A Kaplan-Meier plot illustrating the proportion of men receiving postprostatectomy RT over time and dichotomization around 12 months is shown in eFigure 1 in Supplement 1. The median age for men receiving prostatectomy alone was 60.5 (range, 38.8-79.7) years; prostatectomy plus early RT, 61.8 (range, 44.7-72.9) years; and prostatectomy plus late RT, 60.0 (range, 46.5-74.7) years (*P* = .73). Among the entire cohort, 1075 men (92.0%) were White; Black patients accounted for 68 (6.5%) of men receiving prostatectomy alone; 2 (3.6%) receiving early RT; and 3 (4.8%), receiving late RT. Men receiving early RT vs late or no RT had higher baseline PSA levels, a greater number of positive cores at biopsy, and a greater proportion of Gleason 8 to 10 disease and/or National Comprehensive Cancer Network high-risk disease at the time of prostatectomy (all *P* < .001). Men who received early RT vs late RT were more likely to receive ADT with postprostatectomy RT as well (23 of 57 [40.4%] vs 8 of 64 [12.5%]; *P* < .001). Baseline patient clinicopathologic characteristics by treatment groups are reported in Table 1.

**Table 1.** Patient Clinical and Demographic Characteristics

Characteristic	No. (%)				*P* value^c^
	Early treatment (n = 57)^a^	Late treatment (n = 64)^b^	Only RP (n = 1082)	Total (N = 1203)	
Age, y					
Median (range)	61.8 (44.7-72.9)	60 (46.5-74.7)	60.5 (38.8-79.7)	60.5 (38.8-79.7)	.73
<50	5 (8.8)	2 (3.1)	75 (6.9)	82 (6.82)	NA
50-59	20 (35.1)	30 (46.9)	438 (40.5)	488 (40.57)	
60-69	25 (43.9)	26 (40.6)	484 (44.7)	535 (44.47)	
70-79	7 (12.3)	6 (9.4)	85 (7.9)	98 (8.15)	
Race^d^					
Black	2 (3.6)	3 (4.8)	68 (6.5)	73 (6.25)	.38
White	52 (92.9)	57 (91.9)	966 (92)	1075 (92.0)	
Other^e^	2 (3.6)	2 (3.2)	16 (1.5)	20 (1.71)	
Not reported	1	2	32	35	
BMI, median (range)	27.4 (20-39.5)	27.9 (20.3-44.1)	27.5 (13.4-52.3)	27.6 (13.4-52.3)	.95
College graduate or postgraduate education	23 (40.4)	23 (35.9)	383 (35.4)	429 (35.66)	.75
Married or with partner	6 (10.5)	7 (10.9)	132 (12.2)	145 (12.06)	.96
No. of coexisting illnesses, mean (SD)	1.1 (1.1)	0.9 (1)	0.9 (1)	0.9 (1)	.45
Prostate volume, mean (SD), mL	34.8 (15.5)	40.1 (20.6)	37.7 (16.5)	37.7 (16.7)	.52
PSA at initial diagnosis, ng/mL					
Mean (SD)	10.2 (7.6)	8 (6.2)	6.3 (4.6)	6.6 (5)	<.001
Median (range)	8.3 (2.6-51)	6.1 (1.2-33.4)	5.4 (0.3-71.6)	5.5 (0.3-71.6)	NA
<4	5 (8.8)	10 (15.6)	251 (23.2)	266 (22.11)	
4–10	29 (50.9)	40 (62.5)	713 (65.9)	782 (65)	
>10	23 (40.4)	14 (21.9)	118 (10.9)	155 (12.88)	
Biopsy Gleason score					
<7	12 (21.1)	17 (26.6)	583 (53.9)	612 (50.87)	<.001
7	27 (47.4)	38 (59.4)	447 (41.3)	512 (42.56)	
>7	18 (31.6)	9 (14.1)	52 (4.8)	79 (6.57)	
Clinical category					
T1	36 (63.2)	37 (57.8)	850 (78.7)	923 (76.85)	<.001
T2	21 (36.8)	27 (42.2)	230 (21.3)	278 (23.15)	
% Cores with cancer on biopsy, mean (SD)	61.7 (32.1)	51.5 (30.2)	37.4 (29)	39.2 (29.8)	<.001
Risk tier^f^					
High	21 (36.8)	14 (21.9)	78 (7.2)	113 (9.41)	<.001
Intermediate	28 (49.1)	41 (64.1)	480 (44.4)	549 (45.71)	
Low	8 (14)	9 (14.1)	522 (48.3)	539 (44.88)	

Abbreviations: BMI, body mass index (calculated as weight in kilograms divided by height in meters squared); NA, not applicable; PSA, prostate-specific antigen; RP, radical prostatectomy.

^a^ Early RT, less than 12 months after radical prostatectomy.

^b^ Late RT, 12 months or more after radical prostatectomy.

^c^ *P* values are for the overall comparisons among study groups tested by Kruskal-Wallis test for numerical and Fisher exact test for categorical covariates.

^d^ Race was self-reported.

^e^ Other includes Asian, Indian American, and Native Hawaiian or Pacific Islander.

^f^ The categories of cancer severity are defined as follows: low risk (PSA score <10, Gleason score <7, and T1 category), high risk (Gleason score >7 or PSA score >20), and intermediate risk (all patients who are not at low or high risk).

### HRQOL Outcomes

**Figure 2.**
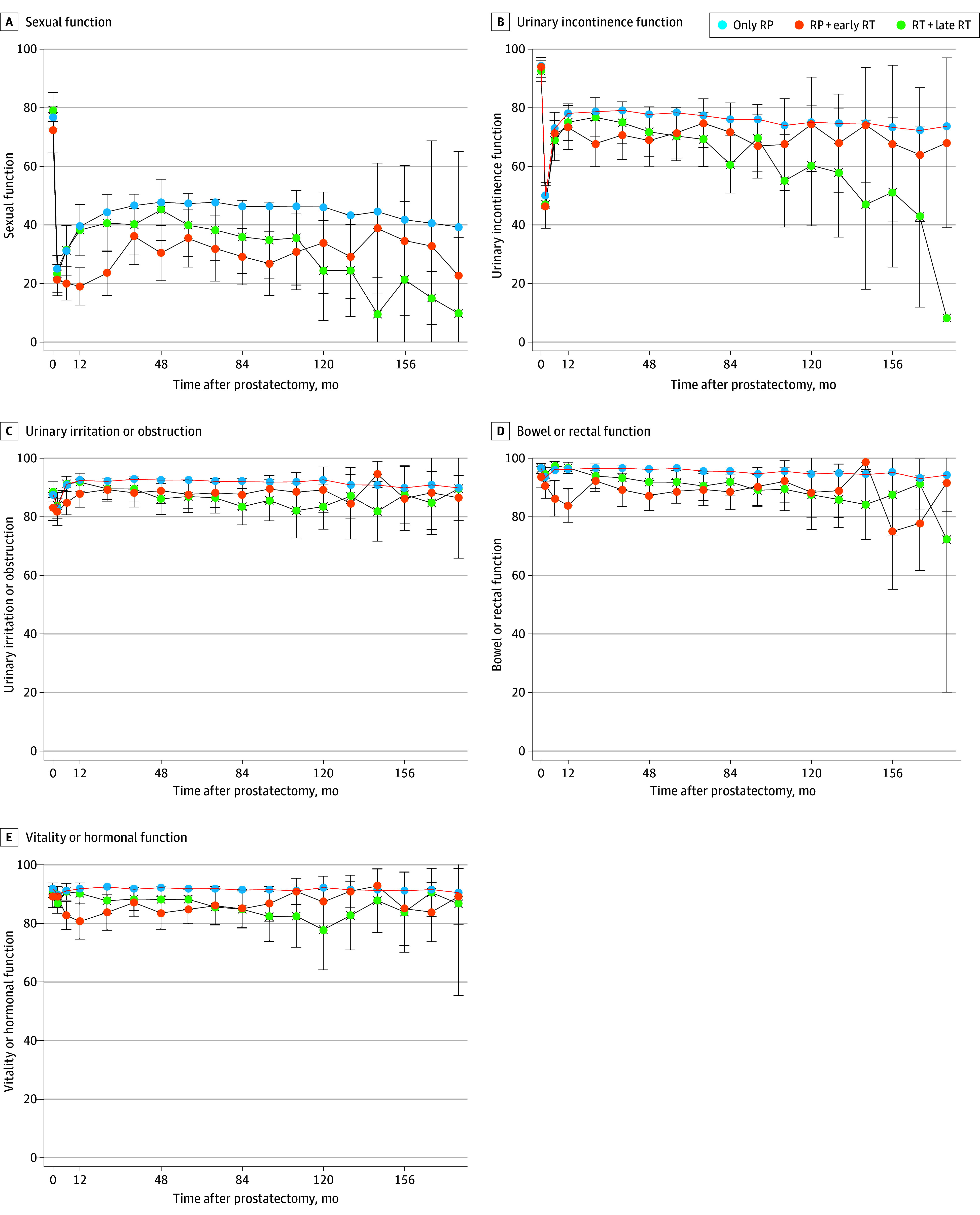
Quality of Life Over Time Among Men Receiving Prostatectomy Alone, Prostatectomy Plus Early Radiation Therapy (RT), or Prostatectomy Plus Late RT The curves show the median; error bars indicate IQRs. Early RT, less than 12 months after radical prostatectomy; late RT, 12 months or more after radical prostatectomy.

Health-related quality of life was evaluated as the change over time in domains of sexual function, urinary incontinence, urinary irritation and/or obstruction, bowel or rectal function, and vitality or hormonal function (Figure 2). Median follow-up time in the entire cohort was 85.6 (IQR, 35.8-117.2) months. The median follow-up time for those receiving no RT was 84.9 (IQR, 35.2-114.0) months; early RT, 97.3 (IQR, 71.0-129.7) months; and late RT, 86.0 (IQR, 54.9-117.6) months. The median follow-up time for PQA and RP2 cohorts was 114.8 months (IQR, 57.9-170.1) for the PQA and 72.6 months (IQR, 24.1-97.7) for the RP2 cohorts.

As previously reported in these cohorts,^11^ significant HRQOL changes in sexual and urinary incontinence domains occurred after prostatectomy, regardless of receipt and/or timing of postprostatectomy RT. Decline in sexual function and urinary incontinence persisted in all cohorts at long-term follow-up beyond 10 years after prostatectomy. Sexual and incontinence symptoms appeared most affected in men who received postprostatectomy RT (early or late) compared with those who received prostatectomy alone.

Multivariable modeling measuring the independent association of receipt of RT and other clinicopathologic covariates with postprostatectomy HRQOL are reported in Table 2. Baseline preprostatectomy domain score and receipt of postprostatectomy RT were associated with a decrease in the HRQOL score for all EPIC-26 domains. Younger age, college education, and nerve-sparing surgery were each associated with changes in sexual scores after prostatectomy. For the urinary incontinence domain, timing of postprostatectomy RT and Black race were associated with a decrease in HRQOL score over time at long-term follow-up. For urinary irritation/obstruction, bowel/rectal function, and vitality/hormonal function, clinicopathologic covariates were associated with change in HRQOL scores after prostatectomy (Table 2). The timing of postprostatectomy RT (early vs late) and pairwise comparison was not associated with change in any HRQOL domain over time.

**Table 2.** Factors Associated With Changes in the Quality-of-Life Score by EPIC Domain After Prostatectomy

Quality-of-life domain and independent variable^a^	*P* value^b^
**Sexuality**	
Receipt of pelvic radiation after prostatectomy	.002^c^
Concurrent ADT	.89
Age	<.001
College education	.001
Nerve-sparing procedure	.001
Pre-RP sexual function	<.001
**Urinary incontinence**	
Receipt of pelvic radiation after prostatectomy	<.00^c^
Concurrent ADT	.69
Black race	.001
Pre-RP urinary incontinence	<.001
**Urinary irritation or obstruction**	
Receipt of pelvic radiation after prostatectomy	<.001^c^
Concurrent ADT	.05
>2 Coexisting illnesses	<.001
Nerve-sparing procedure	<.001
Pre-RP urinary irritation	<.001
**Bowel or rectal function**	
Receipt of pelvic radiation after prostatectomy	<.001^c^
Concurrent ADT	.05
Black race	.005
>2 Coexisting illnesses	<.001
Pre-RP bowel or rectal function	<.001
**Vitality or hormonal function**	
Receipt of RT after prostatectomy	<.001^c^
Concurrent ADT	.02
Age	.03
Black race	<.001
Clinical stage	.04
>2 Coexisting illnesses	<.001
Obesity	.006
Nerve-sparing procedure	.03
Pre-RP hormonal function	<.001

Abbreviations: ADT, androgen deprivation therapy; EPIC, Expanded Prostate Cancer Index Composite; RP, radical prostatectomy; RT, radiotherapy.

^a^ Multivariable mixed linear model identified factors that were associated with changes in health-related quality-of-life over time from baseline (before prostatectomy), while starting model adjusting for the following covariates before variable selection: receipt of RT after prostatectomy (fixed), concurrent ADT and RT (fixed), age at the time of prostatectomy, race, college graduate (yes/no), living with partner (yes/no), aggressive prostate cancer on biopsy (Gleason score >7), prostate volume (milliliters), prostate cancer category T2 (yes/no), number of comorbidities, obesity, nerve-sparing radical prostatectomy, pre-prostatectomy prostate-specific antigen (nanograms per milliliter), and preprostatectomy domain score.

^b^ *P* value depicts independent association across all available time points in multivariable model.

^c^ Pairwise comparison of adjusted difference between early vs late RT is not significant.

The multivariable model was also fitted with a minimally important difference in EPIC-26 domain score as an outcome to support the results. Clinicodemographic features were associated with a clinically significant change in each respective HRQOL domain (eTable 3 in Supplement 1). Timing of postprostatectomy RT in pairwise comparison was associated with long-term bowel function only, with men receiving late RT having a long-term greater decrease than those receiving early RT. Plots showing minimally important differences in HRQOL (higher score indicating greater decrease) are presented in eFigure 2 in Supplement 1.

### HRQOL Changes After Prostatectomy Before Receipt of RT

To measure the possibility of confounding covariates (eg, high-risk disease) independent of RT between treatment cohorts associated with long-term outcomes after prostatectomy, HRQOL was plotted for all patients longitudinally from baseline until the receipt of any RT (eFigure 3 in Supplement 1). There were more substantial postoperative decreases in sexual and vitality/hormonal domains in men who went on to receive RT compared with those who received radical prostatectomy only.

### HRQOL Changes After RT Between Early vs Late RT

**Figure 3.**
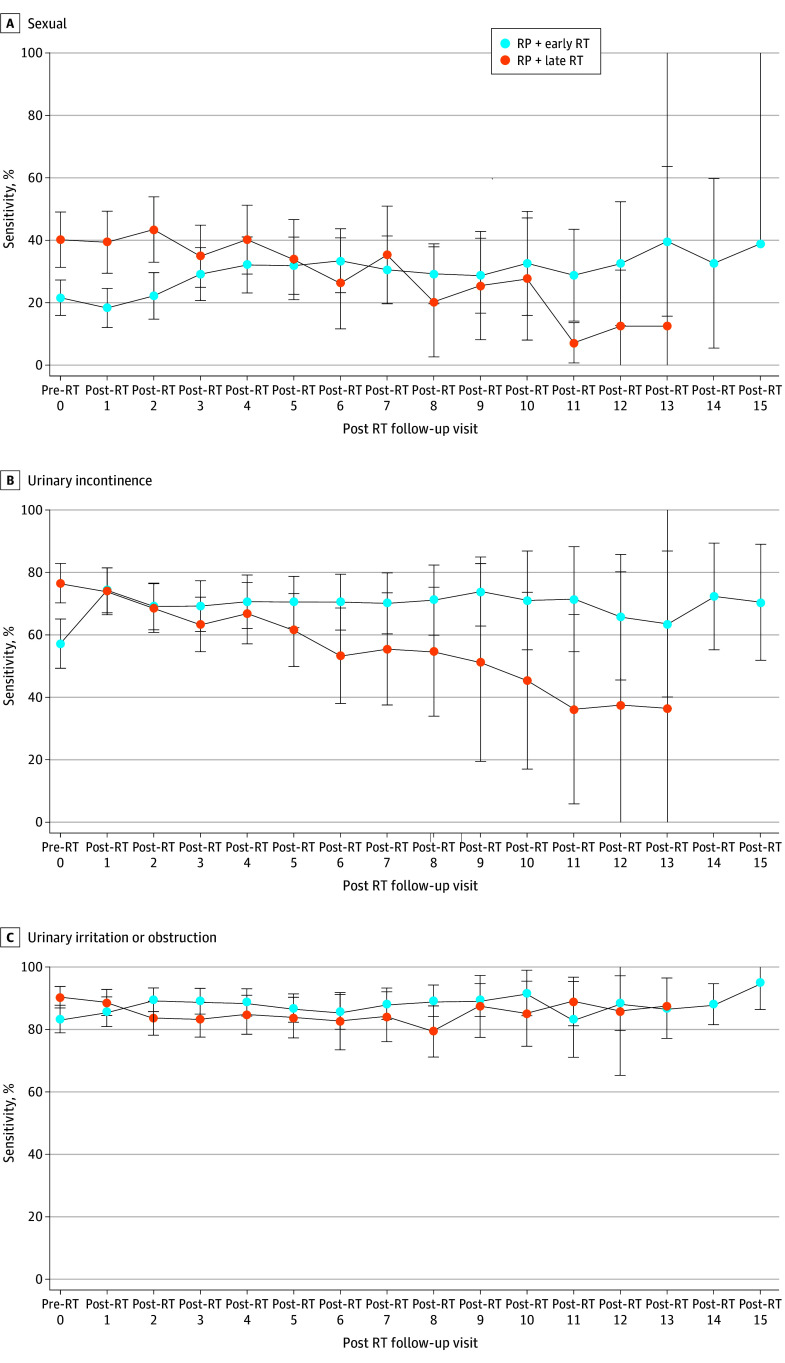
Quality of Life Over Time Centered Around the Time of Radiation Therapy (RT) Among Men Receiving Early vs Late Postprostatectomy RT Error bars indicate IQR. Early RT, less than 12 months after radical prostatectomy; late RT, 12 months or more after radical prostatectomy.

Significant HRQOL differences from immediately before RT to long-term after RT were observed for sexual, incontinence, and urinary irritation between men receiving early vs late RT, as seen in Figure 3. Long-term HRQOL appeared to be improved with early vs late RT. Multivariable modeling measuring RT timing and HRQOL decrease (centered at time of RT) showed RT timing was associated with changes in sexual, incontinence, and urinary irritation domains (eTable 4 in Supplement 1). Given the known HRQOL impact and potential confounding effect of concomitant ADT, long-term HRQOL in early vs late RT, with and without ADT use, was plotted and is shown in eFigure 4 in Supplement 1.

### Incontinence Pad Use Between Early vs Late RT

The proportion of men receiving early vs late RT free from using pads for incontinence over time is shown in eFigure 5 in Supplement 1. Overall, there was a greater proportion of men pad-free after RT among those receiving early compared with late RT. Before the start of postprostatectomy RT, 39.3% of men in the early RT cohort and 73.4% of men in the late RT cohort were pad-free. The proportion of men who achieved freedom from pad use increased over time for those treated with early RT and decreased over time for those treated with late RT. By the sixth visit post-RT, for example, 67.4% of men in the early RT cohort and 47.6% of men in the late RT cohort were pad-free.

## Discussion

In this multi-institutional prospective cohort study of men with localized prostate cancer receiving initial prostatectomy in the PQA and RP2 consortiums, receipt of postprostatectomy RT resulted in decrements in HRQOL scores in all EPIC-26 domains, which persisted at long-term follow-up. The negative outcomes of RT were most notable for sexual and incontinence domains. These findings were not unexpected, especially given that men who received postprostatectomy RT represent a different population than those cured by radical prostatectomy alone, specifically with more aggressive cancer characteristics (eg, 37% high risk in the early RT cohort vs 7% in the radical prostatectomy–only cohort) and frequent use of ADT with RT (eg, 40% use with early RT vs 0% with radical prostatectomy alone). This is particularly highlighted in eFigure 3 in Supplement 1, which shows that men who ultimately received postprostatectomy RT had worse sexual and vitality/hormonal HRQOL decrements after radical prostatectomy, compared with those who received radical prostatectomy alone, even before receiving postprostatectomy RT.

Most notably, however, with a median follow-up of more than 7 years, timing of postprostatectomy RT (ie, <12 vs ≥12 months) was not associated with a decrease in sexual, incontinence, and urinary irritative domain scores at long-term follow-up. Specifically, we observed a greater decrease in sexual and incontinence domain scores in the short-term, within the first 2 years after RT, in men receiving early vs late RT. However, men receiving early RT more frequently received concomitant ADT with RT. Furthermore, men receiving early RT experienced greater recovery of these toxicity domains and achieved similar, and possibly better, domain scores as those receiving late RT at long-term follow-up. For example, when HRQOL domain scores between early and late RT were plotted as a function of time centered around RT delivery, as shown in Figure 3, there was significantly greater recovery of incontinence scores in men receiving early RT compared with those receiving late RT. Additionally, while only 40% of men receiving early RT were pad-free before the RT start, 70% became pad-free at long-term follow-up.

The clinical significance of these findings is that long-term patient-reported outcomes, including erectile function and continence, may not be significantly worsened by early postprostatectomy RT initiated less than 12 months after prostatectomy. This timing cut point is relevant, as adjuvant RT is given soon after prostatectomy when the PSA remains undetectable, often within the first year after surgery.^18^ There remains a subset of men, namely those with adverse pathologic factors and/or genomic classification on prostatectomy specimens, who may have improved outcomes with adjuvant, as opposed to salvage, RT.^10,11^ Yet, 3 randomized clinical trials^6,7^ and a meta-analysis,^9^ on the basis of progression-free survival primarily associated with PSA failure, suggest no major difference when using adjuvant compared with salvage RT. These studies had poor representation of men with adverse pathologic features, including T categories 3 to 4 and Gleason score 8 to 10, and may not be generalizable to this high-risk subgroup. Additionally, these trials did not incorporate genomic classifiers to help risk stratify and identify men who may benefit the most from adjuvant vs salvage RT. Nonetheless, the findings of these studies likely lead most physicians to not offer adjuvant RT to any patient,^19^ despite there being a subset of men who may have inferior outcomes with delayed RT after prostatectomy.^10^ The basis of delaying RT is to mitigate toxic effects, assuming oncologic outcomes are not compromised. However, the findings of our current analysis, using prospective data in 2 rigorously maintained consortiums, suggest no detriment in long-term patient-reported HRQOL outcomes with early vs late RT after prostatectomy.

Our patient-reported toxic effect findings between early and late RT from the PQA and RP2 prospective cohorts are not surprising in the context of those seen in the 3 randomized clinical trials comparing adjuvant vs early salvage RT.^6,7,8^ For example, in the GETUG-AFU 17 trial,^6^ there were no significant differences in patient-reported sexual function, incontinence, urinary irritative symptoms, or bowel symptoms between the 2 arms at 2 and 5 years after RT, as measured by the EORTC prostate cancer–specific QOL questionnaire (QLQ-PR25). In the RADICALS-RT trial,^8^ patient-reported outcome measures for urinary continence and bowel function showed a small but statistically significant worsening of symptoms with adjuvant RT 1 year after randomization, but no significant differences in toxic effects beyond 1 year.

These trials emphasized a detriment in physician-graded toxic effects, primarily confined to grade 1 to 2 bowel or urinary toxic effects, with adjuvant compared with salvage RT. The most prominent differences in toxic effects were observed in the GETUG-AFU 17^6^ and RADICALS-RT^8^ trials, which consistently showed almost a doubling in risk of grade 1 to 2 diarrhea, urinary incontinence, urinary irritation, and hematuria with adjuvant compared with salvage RT. However, within these 2 trials, other factors that could be contributing to these physician-graded toxic effects differences should be highlighted. For example, elective pelvic lymph node RT, which results in higher radiation doses to the bladder and rectum and increased toxic effects,^20^ was at the discretion of the treating clinician for both GETUG-AFU 17 and RADICALS-RT. In RADICALS-RT, for example, 51% of men randomized to adjuvant RT were lymph node–positive or did not receive a pelvic lymph node dissection, compared with 46% of men randomized to salvage RT. Whether men receiving adjuvant RT had higher use of elective pelvic lymph node RT, which could exacerbate acute and late bowel and urinary toxic effects, is unknown. Similarly, use of either 3-dimensional conformal RT (3DCRT) or intensity-modulated RT (IMRT) was allowed; 3DCRT compared with IMRT for pelvic RT is associated with increased genitourinary and gastrointestinal toxic effects.^21^ In GETUG-AFU 17, only 30% of men in the adjuvant RT arm received IMRT, while 47% of men in the salvage RT arm received IMRT. These imbalances in RT delivery technique may have contributed to the significant differences in urinary and bowel toxic effects. In the RAVES randomized trial,^7^ which had the most uniform RT procedures with stringent quality assurance protocols and all patients received prostate-bed RT alone in both arms, grade 2 urinary toxicity was significantly lower with salvage compared with adjuvant RT, but there was no significant difference in gastrointestinal or erectile toxic effects. Nonetheless, since these 3 trials were reported, it has become well accepted that grade 1 to 2 toxicity is expected to be higher with early vs late postprostatectomy RT, especially regarding urinary irritation and incontinence. However, with continued recovery in urinary function, as seen in our long-term analysis, which may obviate any substantial long-term differences, the risks of acute toxic effects with early RT need to be weighed against the potential benefit in outcomes in men with high-risk prostate cancer, adverse features at highest risk of recurrence, and metastasis.

### Limitations

Our study has several limitations. First, participants were accrued from academic, university-affiliated institutions, which may limit the generalizability of our results. However, with growing centralization of prostate cancer care,^22^ as well as multiple studies suggesting superior outcomes in high-volume surgery and RT facilities for men with prostate cancer,^23,24,25,26^ our results gathered from 12 of the highest-volume academic medical centers in the US represent an idealized comparison in settings of high-quality care. Second, this analysis is nonrandomized with respect to receipt and timing of RT, which may introduce imbalances in unmeasurable variables that cannot be accounted for. For example, men receiving prostatectomy alone had more favorable cancer characteristics and did not receive ADT, while men who received postprostatectomy RT had a substantial proportion with high-risk disease and receiving concomitant ADT; these covariates confound the primary outcome and likely attribute to decrements in HRQOL independent of RT. Additionally, men selected for early vs late postprostatectomy RT may have been healthier, with more rapid recovery of continence following surgery and ultimately leading to better long-term functional outcomes. Furthermore, given similar median age between cohorts at the time of initial prostatectomy, men receiving early RT would be younger than men receiving late RT at the time of RT, which may lead to better function recovery. In our analysis, however, baseline HRQOL domain scores did not appear to be substantially different between men receiving early vs late RT, and pre-RT domain scores were accounted for in multivariable analysis. Third, contrary to recent randomized clinical trials,^6,7,8^ our analysis did not directly compare HRQOL between adjuvant vs salvage RT, which typically is determined based on PSA status, pathologic characteristics at surgery, and/or genomic classifiers. Instead, we focused on timing of postprostatectomy RT relative to surgery, independent of whether treatment intent was adjuvant or salvage, to provide an objective, pragmatic analysis of RT timing after surgery and long-term functional outcomes, which may be applicable in the adjuvant or salvage (eg, PSA persistence) setting. Additionally, the dichotomization of early vs late at 12 months, although pragmatic, is reductive and may confound the comparison of this dynamic cohort. For example, men receiving RT at month 2 vs month 11 postprostatectomy (or month 13 vs month 36 within the late RT cohort), may be expected to have different functional outcomes in the long term despite being in the same group. Nonetheless, 12 months was the median RT-free survival of this cohort and remains clinically useful, as most men receiving adjuvant vs salvage postprostatectomy RT initiate treatment within 12 months after surgery.^18^ Fourth, the early and late postprostatectomy RT cohort sizes are relatively small, which limits power to detect statistically significant differences between groups. Fifth, this analysis used prospective data from 2 cohorts, PQA and RP2, separated by time. Men treated in earlier years, especially in the PQA consortium, likely had 3DCRT vs IMRT, which increases the risk of genitourinary and gastrointestinal toxic effects.^21^ The use of IMRT would be expected to mitigate acute and late toxic effects in the postprostatectomy setting, perhaps most pronounced in men receiving early adjuvant RT. The long-term toxic effects data in this cohort are largely dominated by the PQA cohort, which has a median follow-up of 114.8 months, compared with 72.6 months for RP2. As such, a long-term detriment in HRQOL with early postprostatectomy RT may be pronounced due to a predominance of men treated with 3DCRT.

## Conclusions

In this multicenter, longitudinal cohort study using prospective data from the PQA and RP2 consortiums, long-term patient-reported sexual, incontinence, and urinary irritative outcomes did not significantly differ between early vs late postprostatectomy RT; recovery of these HRQOL domains was more pronounced in men receiving early compared with late RT. These results may help guide treatment counseling and support consideration of early RT after prostatectomy for men at particularly high risk of recurrence and metastasis, such as those with PSA persistence or high-risk pathologic and/or genomic features.

## Notes

Supplement 1. **eTable 1.** PQA and RP2 Consortium Institutions **eTable 2.** PROSTQA/RP2 Patient Attrition and Interview Completion Rates **eTable 3.** Factors Associated With Minimally Important Difference by EPIC Domain After Prostatectomy **eTable 4.** Factors Associated With Changes in the Quality-of-Life Score by EPIC Domain After Post-Prostatectomy Radiotherapy Among Patients Who Received Radiation Only **eFigure 1.** Kaplan-Meier Plot of Radiation-Free Survival Amongst the Participants Who Went on to Receive Post-Prostatectomy Radiation Therapy **eFigure 2.** Health-Related Quality of Life Minimally Important Difference Over Time (Higher Score Indicating Greater Decline) **eFigure 3.** Health-Related Quality of Life Over Time From Baseline Up Until the Receipt of Any Radiation Therapy **eFigure 4.** Health-Related Quality of Life Over Time Amongst Men Receiving Early Versus Late Post-Prostatectomy Radiation, With Versus Without Androgen Deprivation Therapy **eFigure 5.** Proportion of Men Free From Urinary Pads Before and After Radiation Amongst Men Receiving Early Versus Late Post-Prostatectomy Radiation Therapy

Supplement 2. **Nonauthor Collaborators.** The PROST-QA/RP2 Consortium

Supplement 3.
Data Sharing Statement
