# Supplementary material for: Postprostatectomy Radiotherapy Timing and Long-Term Health-Related Quality of Life
Source: JAMA Netw Open. 2024 Oct 24;7(10):e2440747. doi: 10.1001/jamanetworkopen.2024.40747 (PMC11581678; doi:10.1001/jamanetworkopen.2024.40747)
Supplement: Supplement 1. — eTable 1. PQA and RP2 Consortium Institutions eTable 2. PROSTQA/RP2 Patient Attrition and Interview Completion Rates eTable 3. Factors Associated With Minimally Important Difference by EPIC Domain After Prostatectomy eTable 4. Factors Associated With Changes in the Quality-of-Life Score by EPIC Domain After Post-Prostatectomy Radiotherapy Among Patients Who Received Radiation Only eFigure 1. Kaplan-Meier Plot of Radiation-Free Survival Amongst the Participants Who Went on to Receive Post-Prostatectomy Radiation Therapy eFigure 2. Health-Related Quality of Life Minimally Important Difference Over Time (Higher Score Indicating Greater Decline) eFigure 3. Health-Related Quality of Life Over Time From Baseline Up Until the Receipt of Any Radiation Therapy eFigure 4. Health-Related Quality of Life Over Time Amongst Men Receiving Early Versus Late Post-Prostatectomy Radiation, With Versus Without Androgen Deprivation Therapy eFigure 5. Proportion of Men Free From Urinary Pads Before and After Radiation Amongst Men Receiving Early Versus Late Post-Prostatectomy Radiation Therapy [file jamanetwopen-e2440747-s001.pdf]

Supplementary Online Content

Patel SA, Patil D, Smith J, et al; PROST-QA/RP2 Consortium. Postprostatectomy radiotherapy timing and long-term health-related quality of life. *JAMA Netw Open*. 2024;7(10):e2440747. doi:10.1001/jamanetworkopen.2024.40747

eTable 1. PQA and RP2 Consortium Institutions

eTable 2. PROSTQA/RP2 Patient Attrition and Interview Completion Rates

eTable 3. Factors Associated With Minimally Important Difference by EPIC Domain After Prostatectomy

eTable 4. Factors Associated With Changes in the Quality-of-Life Score by EPIC Domain After Post-Prostatectomy Radiotherapy Among Patients Who Received Radiation Only

eFigure 1. Kaplan-Meier Plot of Radiation-Free Survival Amongst the Participants Who Went on to Receive Post-Prostatectomy Radiation Therapy

eFigure 2. Health-Related Quality of Life Minimally Important Difference Over Time (Higher Score Indicating Greater Decline)

eFigure 3. Health-Related Quality of Life Over Time From Baseline Up Until the Receipt of Any Radiation Therapy

eFigure 4. Health-Related Quality of Life Over Time Amongst Men Receiving Early Versus Late Post-Prostatectomy Radiation, With Versus Without Androgen Deprivation Therapy

eFigure 5. Proportion of Men Free From Urinary Pads Before and After Radiation Amongst Men Receiving Early Versus Late Post-Prostatectomy Radiation Therapy

This supplementary material has been provided by the authors to give readers additional information about their work.

© 2024 Patel et al. *JAMA Network Open.*

eTable 1: PQA and RP2 Consortium Institutions

PQA (2003-2006)	RP2 (2010-2013)
• Beth Israel Deaconess Medical Center (Boston, MA)	• Beth Israel Deaconess Medical Center (Boston, MA)
• Brigham and Women's Hospital (Boston, MA)	• Brigham and Women's Hospital (Boston, MA)
• Cleveland Clinic (Cleveland, OH)	• Cleveland Clinic (Cleveland, OH)
• Massachusetts General Hospital (Boston, MA)	• Johns Hopkins University (Baltimore, MD)
• MD Anderson Cancer Center (Houston, TX)	• University of Michigan (Ann Arbor, MI)
• University of Michigan (Ann Arbor, MI)	• Michigan State University (East Lansing, MI)*
• Michigan State University (East Lansing, MI)*	• University of California-San Francisco (San Francisco, CA)
• University of California-Los Angeles (Los Angeles, CA)	• Vanderbilt University Medical Center (Nashville, TN)
• Washington University in St. Louis (St. Louis, MI)	• Washington University in St. Louis (St. Louis, MI)

*The Office for Survey Research at Michigan State University administered the third-party phone interviews collecting patient-reported outcome measures for both cohorts

© 2024 Patel et al. *JAMA Network Open.*

eTable 2: PROSTQA/RP2 Patient Attrition and Interview Completion Rates

Study Time-point	No. of patients	No. of patients who completed the interview
Before Treatment	1203	1203
Follow-up month 2	1203	1137
Follow-up month 6	1191	1122
Follow-up month 12	1179	1077
Follow-up month 24	1149	974
Follow-up month 36	1121	857
Follow-up month 48	1094	848
Follow-up month 60	1074	763
Follow-up month 72	1059	679
Follow-up month 84	1052	630
Follow-up month 96	1044	566
Follow-up month 108	1040	384
Follow-up month 120	509	283
Follow-up month 132	504	268
Follow-up month 144	500	223
Follow-up month 156	492	213
Follow-up month 168	468	180
Follow-up month 180	401	142

© 2024 Patel et al. *JAMA Network Open.*

eTable 3: Factors Associated with Minimally Important Difference by EPIC domain after Prostatectomy

Quality-of-Life Domain	Independent Variable[a]	P-value[b]
**Sexuality**	Receipt of Pelvic Radiation After Prostatectomy	0.004[c]
	Concurrent ADT	0.92
	Age	<0.001
	College Education	0.001
	Nerve-Sparing Procedure	0.000
	Pre-RP Sexual Function	<0.001
**Urinary incontinence**	Receipt of Pelvic Radiation After Prostatectomy	<0.001[c]
	Concurrent ADT	0.76
	Black race	0.001
	Pre-RP Urinary Incontinence	<0.001
**Urinary irritation or obstruction**	Receipt of Pelvic Radiation After Prostatectomy	<0.001[c]
	Concurrent ADT	0.05
	Black race	0.04
	>2 Coexisting illnesses	0.001
	Nerve-Sparing Procedure	0.001
	Pre-RP Urinary Irritation	<0.001
**Bowel or rectal function**	Receipt of Pelvic Radiation After Prostatectomy	<0.001[d]
	Concurrent ADT	0.06
	Black race	0.005
	>2 Coexisting illnesses	<0.001
	Pre-RP Bowel or Rectal Function	<0.001
**Vitality or hormonal function**	Receipt of Pelvic Radiation After Prostatectomy	<0.001[c]
	Concurrent ADT	0.03
	Age	0.03
	Black race	0.000
	>2 Coexisting illnesses	<0.001
	Obesity	0.003
	Nerve-Sparing Procedure	0.02
	Pre-RP Hormonal Function	<0.001

a. Multivariable mixed linear model identified factors that were significantly associated with Minimally Important Difference HRQOL over time from baseline (prior to prostatectomy), while starting model adjusting for following covariates before variable selection: Receipt of radiation after prostatectomy (fixed), Concurrent ADT with RT (fixed), Age at the time of prostatectomy, Patient reported race, college graduate (Yes/No), living with partner (Yes/No), Aggressive prostate cancer on biopsy (GS7+), Prostate Volume (mL), Prostate cancer Stage T2 (Yes/No), Number of comorbidities, Obesity, Nerve-sparing radical prostatectomy, Pre-prostatectomy PSA (ng/mL), Pre-prostatectomy Domain score.
b. p-value depicts independent association across all available time-points in multivariable model
c. Pairwise comparison of adjusted difference between early vs late radiation is not significant
d. Pairwise comparison of adjusted difference between early vs late radiation is significant

© 2024 Patel et al. *JAMA Network Open.*

eTable 4: Factors Associated with Changes in the Quality-of-Life Score by EPIC domain after post-prostatectomy radiotherapy among patients who received radiation only

Quality-of-Life Domain	Independent Variable[a]	P-value[b]
**Sexuality**	Time of Radiation After Prostatectomy	0.02
	Concurrent ADT	0.003
	Age	<0.001
	Biopsy Gleason score ≥ 7	0.003
	Pre-radiation Sexual Function	<0.001
**Urinary incontinence**	Time of Radiation After Prostatectomy	0.003
	Concurrent ADT	0.09
	College Education	0.03
	Living with partner	0.02
	Obesity	0.03
**Urinary irritation or obstruction**	Time of Radiation After Prostatectomy	0.004
	Concurrent ADT	0.80
**Bowel or rectal function**	Time of Radiation After Prostatectomy	0.10
	Concurrent ADT	0.23
	>2 Coexisting illnesses	0.02
	Pre-radiation Bowel or Rectal Function	<0.001
**Vitality or hormonal function**	Time of Radiation After Prostatectomy	0.09
	Concurrent ADT	0.10
	Biopsy Gleason score ≥ 7	0.007
	PSA	0.01

a. Multivariable mixed linear model identified factors that were significantly associated with changes in HRQOL over time from baseline (nearest visit prior to post-prostatectomy radiotherapy) to end of follow-up, while starting model adjusting for following covariates before variable selection: Time to start of radiation after prostatectomy (fixed), Concurrent ADT with RT (fixed), Age at the time of prostatectomy, Race, College graduate (Yes/No), Living with partner (Yes/No), Aggressive prostate cancer on biopsy (GS7+), Prostate Volume (mL), Prostate cancer Stage T2 (Yes/No), Number of comorbidities, Obesity, Nerve-sparing radical prostatectomy, Pre-prostatectomy PSA (ng/mL), Domain score at baseline as defined above.
b. p-value depicts independent association across all available time-points in multivariable model

© 2024 Patel et al. *JAMA Network Open.*

eFigure 1: Kaplan-Meier plot of radiation-free survival amongst the participants who went on to receive post-prostatectomy radiation therapy

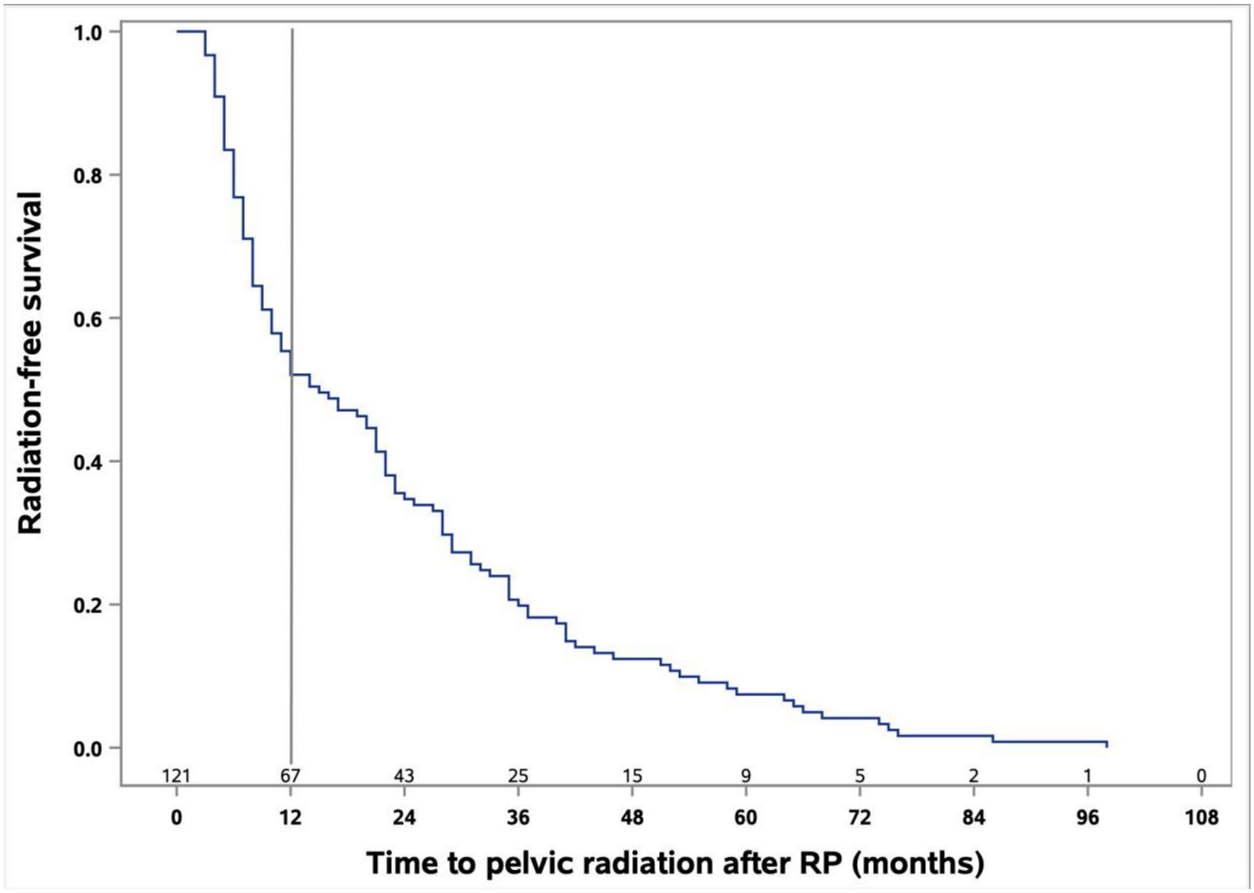

© 2024 Patel et al. *JAMA Network Open.*

eFigure 2: Health-related quality of life minimally important difference over time (higher score indicating greater decline)

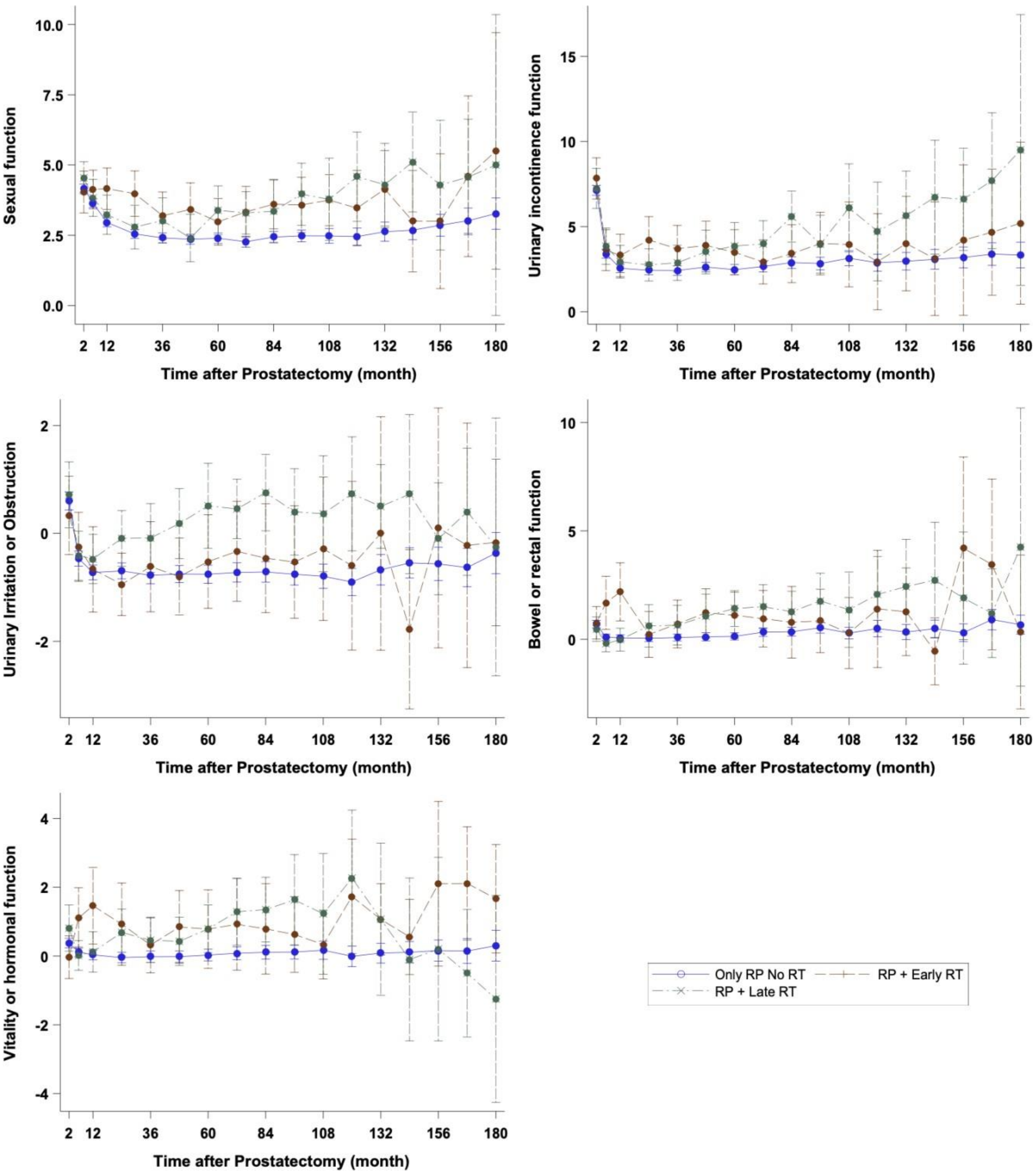

© 2024 Patel et al. *JAMA Network Open.*

eFigure 3: Health-related quality of life over time from baseline up until the receipt of any radiation therapy

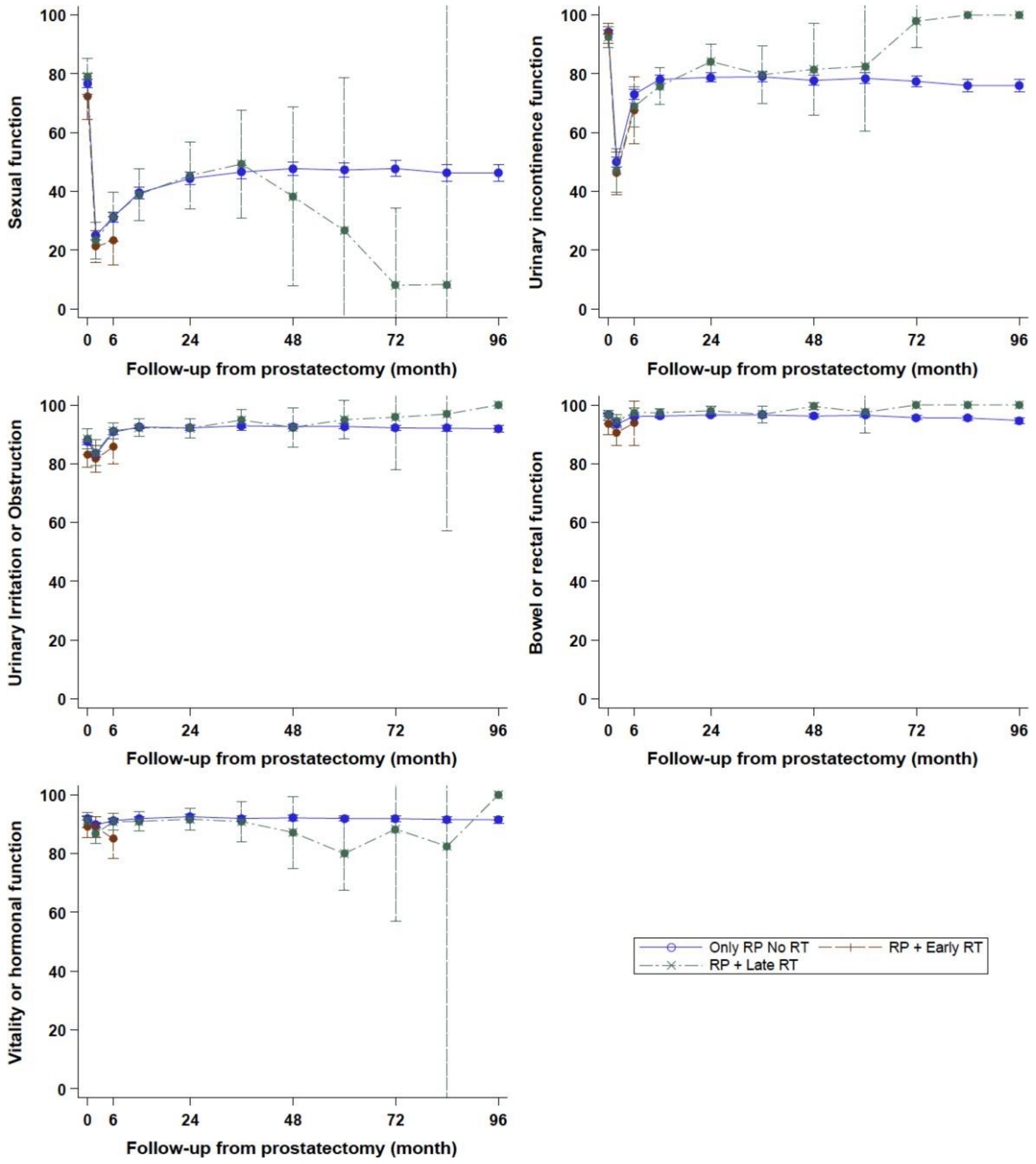

© 2024 Patel et al. *JAMA Network Open.*

eFigure 4: Health-related quality of life over time amongst men receiving early versus late post-prostatectomy radiation, with versus without androgen deprivation therapy

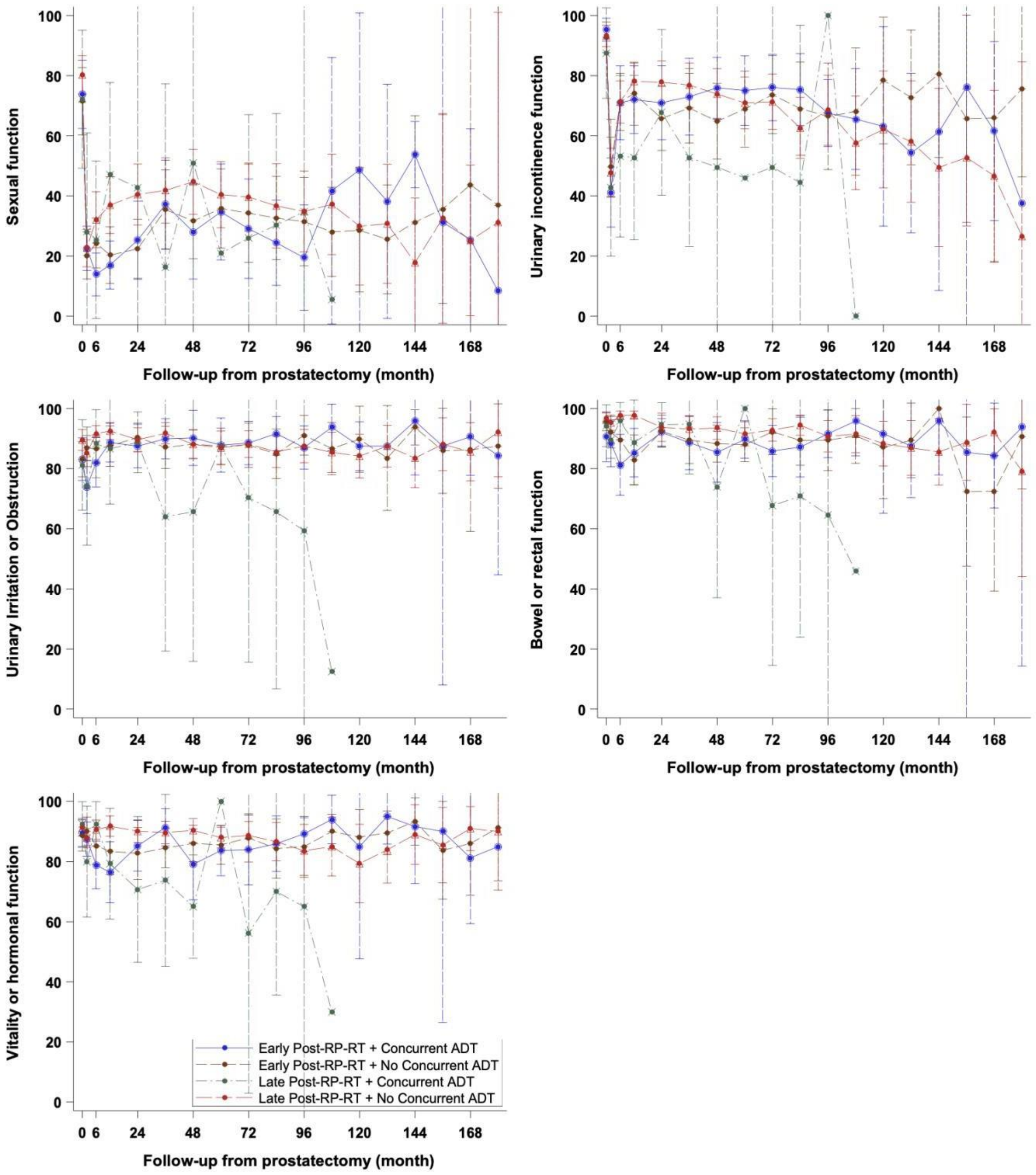

© 2024 Patel et al. *JAMA Network Open.*

eFigure 5: Proportion of men free from urinary pads before and after radiation amongst men receiving early versus late post-prostatectomy radiation therapy

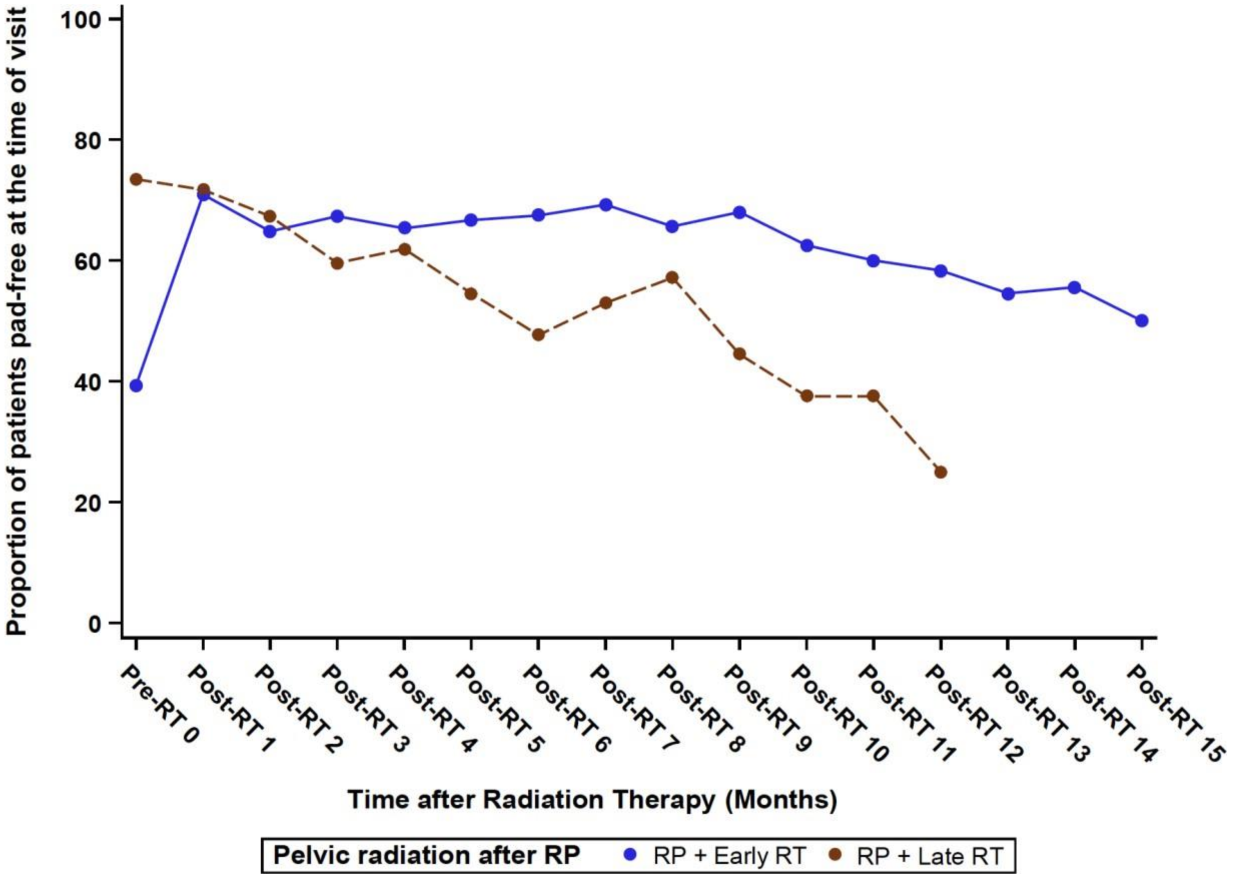

© 2024 Patel et al. *JAMA Network Open.*
