# Supplementary material for: Postprostatectomy Radiotherapy Timing and Long-Term Health-Related Quality of Life
Source: JAMA Netw Open. 2024 Oct 24;7(10):e2440747. doi: 10.1001/jamanetworkopen.2024.40747 (PMC11581678; doi:10.1001/jamanetworkopen.2024.40747)
Supplement: Supplement 2. — Nonauthor Collaborators. The PROST-QA/RP2 Consortium [file jamanetwopen-e2440747-s002.pdf]

Supplemental Online Content: Nonauthor Collaborators
*First name, last name, and suffix (if applicable) are required and will appear in PubMed.

***Group Name(s):**							
***First Name and Middle Initial(s)**	***Last Name**	***Suffix (eg, Jr, III)**	Academic Degrees	Institution	Location (city, state/province, country)	Role or Contribution, eg, chair, principal investigator	Group (if more than 1 Group listed in the byline) and/or Subgroup (eg, Steering Committee)
Rodney	Dunn		MS	University of Michigan	Ann Arbor, MI		
Laurel	Northouse		PhD, RN	University of Michigan	Ann Arbor, MI		
Thomas	Greenfield		PhD	Alcohol Research Group, Public Health Institute	Emeryville, CA		
Jay	Ciezki		MD	Cleveland Clinic	Cleveland, OH		
Douglas	Dahl		MD	Massachusetts General Hospital	Boston, MA		
Anthony	Zietman		MD	Massachusetts General Hospital	Boston, MA		
Felix	Feng		MD, PhD	University of California, San Francisco	San Francisco, CA		
Ted	Skolarus		MD	University of Chicago	Chicago, IL		
Kyle	Davis		BS	Michigan State University	East Lansing, MI		
Linda	Stork		BS	Michigan State University	East Lansing, MI		
Arul	Mahadevan		MD	Massachusetts General Hospital	Boston, MA		
