# Supplementary material for: Postprostatectomy Radiotherapy Timing and Long-Term Health-Related Quality of Life
Source: JAMA Netw Open. 2024 Oct 24;7(10):e2440747. doi: 10.1001/jamanetworkopen.2024.40747 (PMC11581678; doi:10.1001/jamanetworkopen.2024.40747)
Supplement: Supplement 3. — Data Sharing Statement [file jamanetwopen-e2440747-s003.pdf]

Data Sharing Statement

Patel. Postprostatectomy Radiotherapy Timing and Long-Term Health-Related Quality of Life. *JAMA Netw Open*. Published October 24, 2024. doi:10.1001/jamanetworkopen.2024.40747

Data
Data available: No
